# Efficacy of Alkaline-Treated Soy Waste Biomass for the Removal of Heavy-Metal Ions and Opportunities for Their Recovery

**DOI:** 10.3390/ma14237413

**Published:** 2021-12-03

**Authors:** Laura Bulgariu, Daniela Ionela Ferţu, Irina Gabriela Cara, Maria Gavrilescu

**Affiliations:** 1 Department of Environmental Engineering and Management, “Cristofor Simionescu” Faculty of Chemical Engineering and Environmental Protection, Gheorghe Asachi Technical University of Iaşi, 700050 Iaşi, Romania; 2 Department of Pharmaceutical Sciences, Faculty of Medicine and Pharmacy, Dunarea de Jos University of Galaţi, 800002 Galati, Romania; danafertu2004@yahoo.com; 3 Research Institute for Agriculture and Environment, “Ion Ionescu de la Brad” Iasi University of Life Sciences, 700490 Iasi, Romania; carairina@uaiasi.ro; 4 Academy of Romanian Scientists, 54 Splaiul Independentei, 050094 Bucharest, Romania

**Keywords:** soy waste biomass, alkaline treatment, biosorption, heavy-metal ions, metal recovery

## Abstract

In this study, soy waste biomass (SW) resulting from oil extraction was treated with alkaline solution, and the obtained material (Na-SW) was used as biosorbent for the removal of Pb(II), Cd(II), and Zn(II) ions from aqueous media. The performance of this biosorbent was examined in batch systems, at different initial metal ion concentrations and contact times (pH 3.4; 5 g of biosorbent/L). Isotherm and kinetic modeling was used to calculate the equilibrium and kinetics of the biosorption processes. The maximum biosorption capacity, calculated from the Langmuir isotherm model, followed the order Zn(II) (0.49 mmol/g) > Cd(II) (0.41 mmol/g) ≈ Pb(II) (0.40 mmol/g), while the kinetics of biosorption processes fit the pseudo-second-order model. Three cycles of biosorption/desorption were performed to estimate the reusability of Na-SW biosorbent, and the regeneration efficiency was higher than 97% in all cases. The practical applicability of Na-SW biosorbent in treating of wastewater contaminated with Pb(II), Cd(II), and Zn(II) ions was examined using simulated wastewater samples, and the main quality characteristics of the effluents obtained after treatment were evaluated. All these aspects highlight the potential applicability of Na-SW for large-scale wastewater treatment.

## 1. Introduction

Heavy-metal pollution is currently considered one of the most important environmental concerns worldwide. This is due to the fact that many industrial activities use large quantities of heavy metals, and the disposal of such industrial effluents, without proper treatment, contributes significantly to environmental pollution [1,2,3]. Moreover, once the contaminated industrial wastewater is discharged into the environment, heavy metals do not degrade and have an accumulating tendency, causing many health problems [4,5,6].

Heavy metals such as lead, cadmium, and zinc occur frequently in industrial wastewater, in significant quantities, due to their industrial importance in various production sectors (i.e., mining, electroplating, galvanization, battery manufacturing, fertilizer production, etc.) [7,8,9]. These metal ions are chemically stable, have high mobility, and can remain in the environment for a long time, causing pollution. On the basis of these considerations, it is essential to control and reduce the heavy-metal content of industrial effluents before they are discharged into the environment.

To remove heavy-metal ions from aqueous media, several conventional methods, such as precipitation, ion exchange, reverse osmosis, and coagulation [10,11,12,13,14], are used on an industrial scale. However, most of these methods have high costs, require high energy, and produce large quantities of toxic sludge, which makes them not environmentally friendly [7,15]. In comparison, biosorption is a promising method for removing heavy-metal ions from aqueous effluents, because it is facile, low-cost, and efficient [2,7,16,17]. Thus, various types of biomass, such as algae, microorganisms, aquatic plants, agricultural waste and byproducts [18,19,20], have been reported in the literature for the efficient removal of heavy metals.

However, all the advantages of biosorption are valid only if the material used as biosorbent is easy to obtain and can retain large amounts of heavy-metal ions [21,22]. The first requirement is mainly related to the availability of the biosorbent and the number of preparation stages [23,24]. This requirement can be fulfilled if biomass wastes from other industrial activities are used as biosorbents, because (i) these materials are already available in large quantities, being necessary in industrial activities, (ii) some preparation stages, such as washing, preliminary drying, grinding, and sizing, are already done, (iii) their purchase price is low because they are considered waste from industrial activities, and (iv) in addition to the benefits related to environmental protection, it is possible to obtain products with added value in accordance with the principles of the circular economy. Thus, numerous types of waste biomasses from the agriculture, food, fermentation, and drug industries [19,22,23,24] have been used as biosorbents for the removal of various heavy-metal ions from aqueous effluents (such as Pb(II), Cd(II), Cu(II), Co(II), and Zn(II)), to find new alternatives for the cleaning of contaminated industrial effluents before discharge into the environment. Much lower attention has been paid to biomass waste from the biofuel production industry. This is because such waste contains traces of organic solvents (used for oil extraction) in its composition and is considered hazardous. Therefore, finding a simple and cheap way to remove traces of organic solvents from the composition of biomass waste from the biofuel industry is a challenge that must be overcome in order to use these wastes as biosorbents. According to studies from the literature [25,26], the removal of traces of organic solvents from waste biomass composition after oil extraction can be done in two different ways: (i) heating the biomass waste at a certain temperature to allow evaporation of the organic solvent, and (ii) treatment of the waste biomass with alkaline solution (such as NaOH), in certain experimental conditions, followed by washing of the obtained biosorbent. The heating of waste biomass is undesirable in the preparation of biosorbents, because the removal of the traces of organic solvents often requires rather high temperatures or successive heating stages, which can lead to the destruction of functional groups on the biomass surface, thus lowering its biosorptive performance [27,28]. More suitable from this perspective is the treatment of waste biomass with alkaline solutions. The treatment of waste biomass from biofuel production with alkaline solution (such as NaOH) has three major advantages: (i) the hydrophobicity of the waste biomass surface is decreased, which makes the removal of traces of organic solvents much easier (this is the reason why NaOH solutions are used, whose caustic effect is more pronounced), without the need for heating at high temperatures; (ii) such treatments can be done at ambient conditions and, thus, the cost of the biosorbent preparation remains low; (iii) the dissociation degree of superficial functional groups from the waste biomass surface is increased, which results in an increase in the efficiency of the obtained materials in the biosorption processes of the metal ions [29]. Therefore, in addition to the fact that the alkaline treatment helps to eliminate the traces of organic solvents from the composition of such biomass waste, it also improves the biosorptive performances of the obtained materials, thus satisfying the second requirement mentioned above.

Starting with all these observations, in this study, soy waste biomass (SW) resulting from oil extraction was treated with alkaline solution (NaOH) and used as biosorbent (Na-SW) for the removal of Pb(II), Cd(II), and Zn(II) ions from aqueous media. The biosorptive performance of Na-SW was analyzed in batch systems, at different initial concentrations of heavy-metal ions and contact times. Three cycles of biosorption/desorption were performed to estimate the reusability of Na-SW in the removal processes of heavy metals. The utility of Na-SW biosorbent in treating wastewater contaminated with Pb(II), Cd(II), and Zn(II) ions was examined using artificial wastewater samples. Moreover, a future research plan for the recovery of metal ions from industrial effluents is presented to highlight the practical applicability of this study on a large scale.

## 2. Materials and Methods

### 2.1. Metal Ion Solutions and Measurements

Stock solutions (10^−2^ mol M(II)/L) of Pb(II), Cd(II), and Zn(II) ions were prepared using metal nitrate salts (from Aldrich) in distilled water. Each working solution was obtained from the stock solutions. Prior to the biosorption experiments, the pH was adjusted to 3.4 (optimal value) using a 10^−2^ M HNO_3_ solution (purchased from Chemical Company, Iaşi, Romania). Initial and equilibrium concentrations (after sample filtration) of each heavy-metal ion were analyzed spectrophotometrically (Digital Spectrophotometer UV-VIS Cary 60 (Agilent, New York, NY, USA, 1 cm glass cell), using a specific method (Table 1).

### 2.2. Biosorbent Preparation and Characterization

Soy waste biomass (SW) was obtained from ground soybeans (commercial available on the Romanian market) after oil extraction with *n*-hexane for 24 h. The resultant waste biomass was dried in air (50 ± 1 °C) for 24 h. The alkaline-treated soy waste biomass (Na-SW) was obtained by treating 5 g of soy waste biomass with 100 mL of 0.1 N NaOH solution, for 24 h at room temperature (22 ± 1 °C). After filtration, Na-SW was washed with distilled water (until neutral pH), dried in air (50 ± 1 °C), and mortared. The changes in functional groups on the surface of soy waste biomass, before and after alkaline treatment or before and after heavy-metal ion biosorption, were highlighted by recording the FTIR spectra (Bio-Rad FTIR spectrometer, Berlin, Germany), spectral domain = 400–4000 cm^−1^, resolution = 4 cm^−1^, 32 scans, KBr pellt method). The surface morphology of the biosorbent was examined by scanning electron microscopy (Tokio, Japan)(SEM Hitachi S 3000 N), at different magnification.

### 2.3. Biosorption Experiments

The biosorption experiments were performed by batch technique, mixing 0.125 g of Na-SW with 25 mL of solution, containing various concentrations of Pb(II), Cd(II), and Zn(II) ions (10–420 mg M(II)/L), for different contact times (5–180 min), at a constant initial solution pH of 3.4 and room temperature (22 ± 1 °C). In each case, at the end of biosorption procedure, the phases were separated by filtration, and the concentration of metal ions in filtrate was analyzed spectrophotometrically (Table 1). The values of equilibrium concentration were then used for the calculation of the biosorption parameters (*q*, mg/g and *R*, %), using Equations (1) and (2).

(1) $$q=\frac{\left(c_{0}-c)\cdot (V/1000\right)}{m},$$

(2) $$R=\frac{c_{0}-c}{c_{0}}\cdot 100,$$

where *c_0_* and *c* are the initial and equilibrium concentrations of heavy-metal ions in solution (mg/L), *V* is the volume of solution (mL), and *m* is the mass of biosorbent (g).

The same sample of biosorbent was then used in three biosorption/desorption successive cycles. For biosorption, 1 g of Na-SW was treated with 100 mL of each heavy-metal ion solution (60–100 mg/L) at pH 3.4. After 3 h, each Na-SW sample loaded with metal ions was filtered, washed three times with 10 mL of distilled water, and dried in air. For desorption, 0.1 g of Na-SW loaded with metal ions was treated with 10 mL of a 10^−2^ N HNO_3_ solution, stirred intermittently for 3 h, and then filtered. The heavy-metal ion concentration after each desorption cycle was analyzed spectrophotometrically as described above (Table 1).

To test the applicability of Na-SW in the biosorption processes, three samples of 250 mL of artificial wastewater were prepared using tap water and stock solutions of heavy metals, and then used for the biosorption experiments. The pH of each wastewater sample was adjusted to 3.4, and then 1.25 g of Na-SW was added. After 3 h, the samples were filtered, and the heavy-metal ion concentrations, as well as other quality parameters, were analyzed using standard procedures [31].

## 3. Results and Discussion

### 3.1. Structural Characteristics of Na-SW Biosorbent

**Figure 1:**
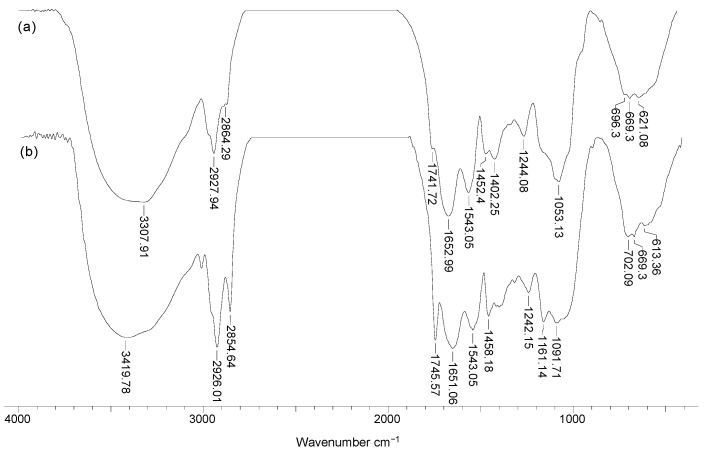
FTIR spectra of soy waste biomass before (**a**) and after (**b**) alkaline treatment.

To highlight the structural particularities which can play an important role in the biosorption processes, FTIR spectra and SEM images were recorded for soy waste biomass before and after alkaline treatment. FTIR spectra (Figure 1) clearly show that, compared with SW biomass (spectrum a), Na-SW biosorbent (spectrum b) had more superficial functional groups (hydroxyl, carboxyl, carbonyl, esteric, etheric, etc.), since the absorption bands at 3419, 1745, 1458, 1161, and 1091 cm^−1^ had a higher intensity and were shifted to higher wavelengths.

The increased intensity of these absorption bands suggests that, on the surface of Na-SW, the number of available functional groups was higher compared with SW biomass. In addition, the shift of absorption bands to higher wavelengths indicates that these functional groups had more degrees of freedom, probably due to the breakage of physical (hydrogen) bonds, thus facilitating interaction with metal ions in aqueous solutions.

On the other hand, the disappearance of the absorption band at 2864 cm^−1^, which is characteristic to *n*-hexane [30], shows that the alkaline treatment successfully removed traces of organic solvent used in oil extraction. Therefore, the risk of contamination of aqueous effluents treated by biosorption using this biosorbent was significantly reduced.

**Figure 2:**
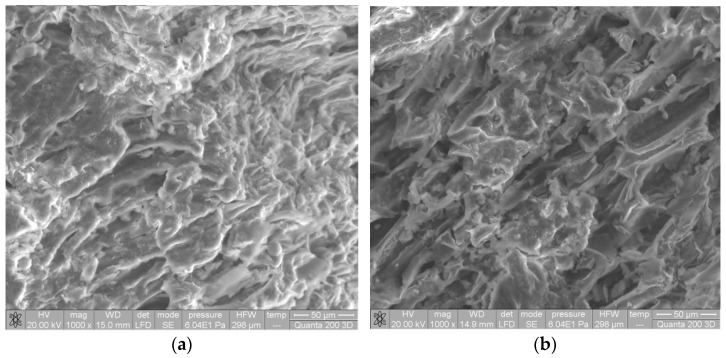
SEM images of soy waste biomass before (**a**) and after (**b**) alkaline treatment.

Significant changes can also be observed in the morphology of the biosorbent surface after alkaline treatment (Figure 2). Thus, after alkaline treatment, the surface of SW biomass became much more irregular and wrinkled, with much better defined pores. The higher availability of functional groups (proven by FTIR spectra) and the higher surface porosity (proven by SEM images) are two important features that highlight the possible use of Na-SW as a biosorbent for removing heavy-metal ions from aqueous environments.

### 3.2. Effect of Initial Heavy-Metal Ion Concentration and Isotherm Modeling

**Figure 3:**
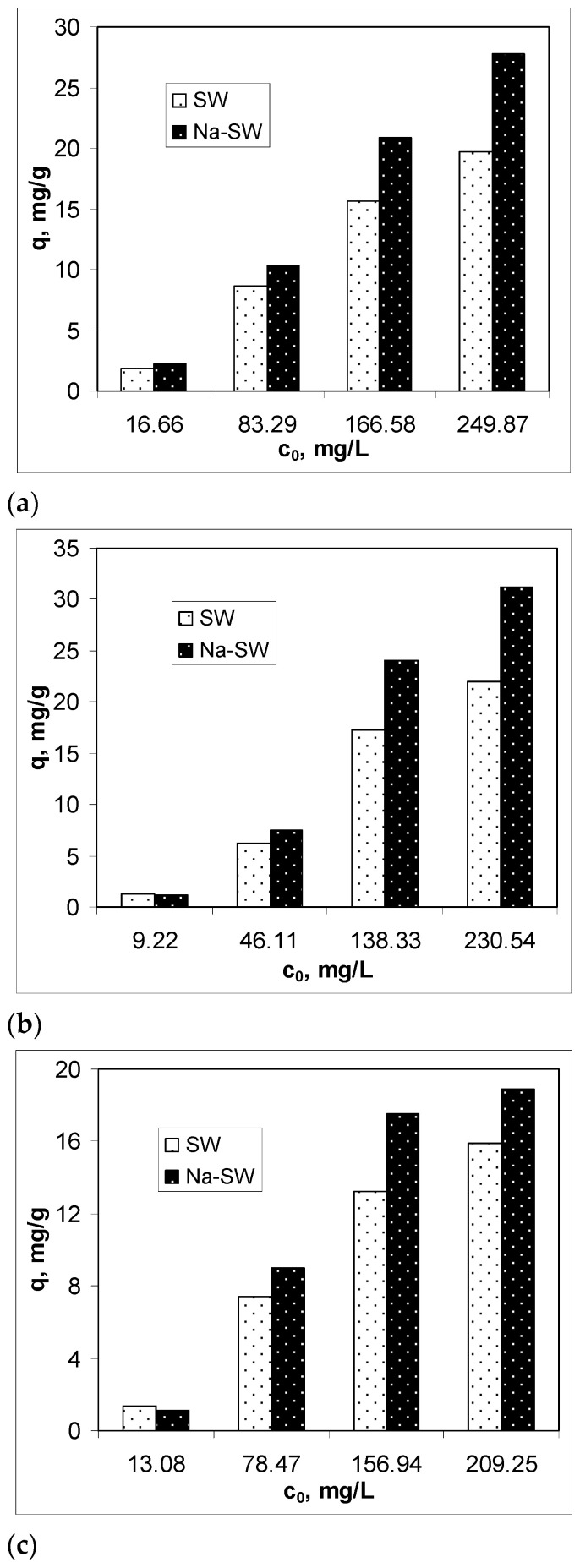
Comparison of biosorption capacities of SW and Na-SW for different initial concentrations of Pb(II) (**a**), Cd(II) (**b**), and Zn(II) (**c**).

To examine the efficiency of Na-SW biosorbent in removing heavy-metal ions (Pb(II), Cd(II), and Zn(II)) from aqueous solution, the biosorption capacity was determined at different initial metal ion concentrations, between 10 and 250 mg M(II)/L, at an initial solution pH of 3.4, using 5 g of biosorbent/L at room temperature (22 ± 1 °C), in comparison with untreated soy waste biomass (SW). The obtained results are illustrated in Figure 3.

The experimental results presented in Figure 3 highlight two important aspects. First, the biosorption capacity of Na-SW depended on the initial heavy-metal ion concentration and increased with the increase in this parameter as follows: Pb(II) > Cd(II) > Zn(II). This variation suggests that, on the surface of Na-SW, there are sufficient functional groups that this biosorbent is efficient even at high initial concentrations of metal ions. Second, in the low initial concentration range, the biosorption capacities of SW and Na-SW were comparable, whereas, in the high initial concentration range, Na-SW proved its higher biosorption capacity for all studied metal ions compared with SW, and these differences were greater as the initial concentration of heavy-metal ions increased. Therefore, it can be said that, after the alkaline treatment, more functional groups became available for interaction with the heavy-metal ions in the aqueous solution, thus increasing the efficiency of this biosorbent. Specifically, for the lowest initial metal ion concentrations, the increase in biosorption capacity was 16.86% in the case of Pb(II), 19.19% in the case of Cd(II), and 8.95% in the case of Zn(II), whereas, for the higher initial metal ion concentrations, the increase in biosorption capacity was 44.26% for Pb(II), 39.07% for Cd(II), and 26.29% for Zn(II), compared with SW biomass.

**Figure 4:**
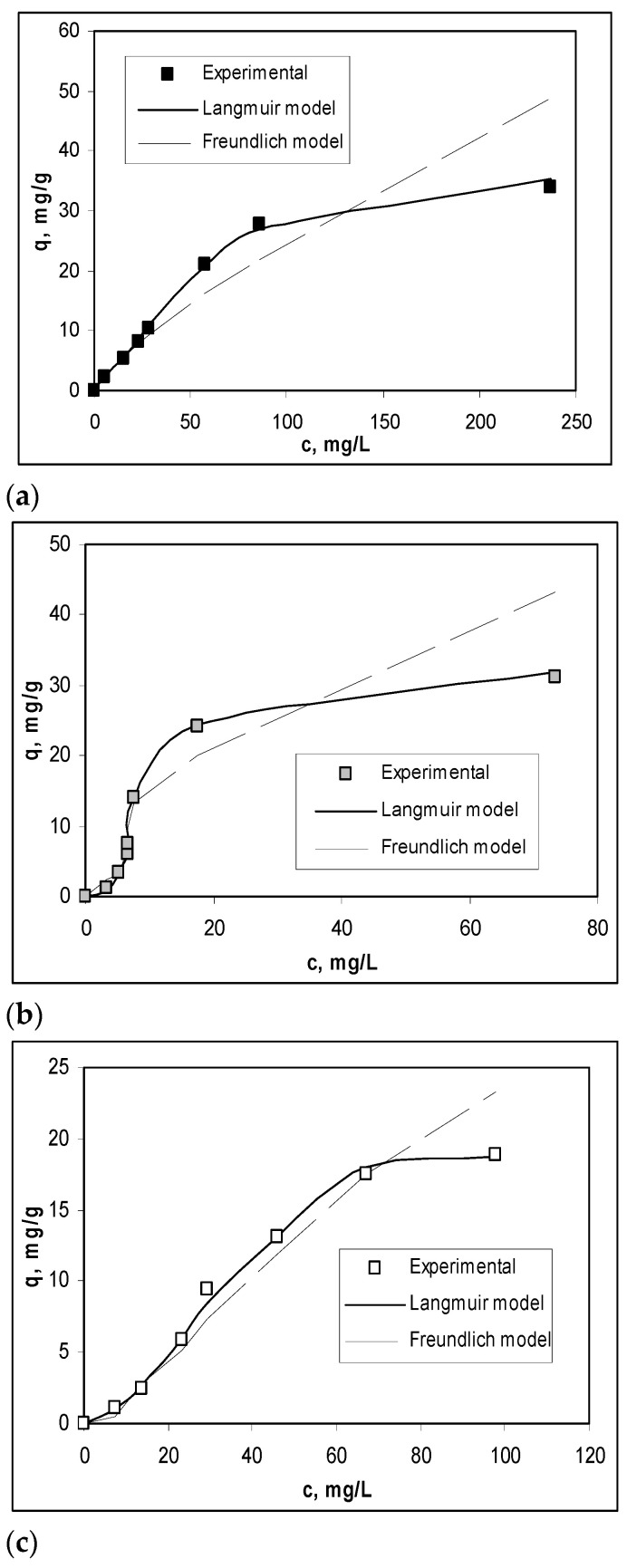
Experimental and modeled isotherms obtained for the biosorption of Pb(II) (**a**), Cd(II) (**b**), and Zn(II) (**c**) on Na-SW biosorbent.

To obtain a quantitative evaluation of the efficiency of Na-SW biosorbent in the biosorption processes of Pb(II), Cd(II), and Zn(II) ions, the experimental isotherms were modeled using Langmuir and Freundlich isotherm models. The overlapping of the experimental isotherms with those obtained by modeling is shown in Figure 4, while the parameters characteristic of each model are summarized in Table 2.

As shown in Figure 4 and Table 2, the Langmuir isotherm model best fit the experimental data (*R^2^* > 0.99), indicating a monolayer biosorption of heavy-metal ions on Na-SW biosorbent. The maximum biosorption capacity (*q*_max_, mg/g) increased in the order Zn(II) (0.49 mmol/g) > Cd(II) (0.41 mmol/g) ≈ Pb(II) (0.40 mmol/g), which is similar to the variation in ionic radius of these ions, showing that the retention of heavy-metal ions occurs at the surface of the biosorbent [32]. In addition, the very close values of Langmuir constant (Table 2) suggest that the biosorption processes involve the same types of interactions between functional groups from the Na-SW biosorbent surface and metal ions, and these interactions are most likely electrostatic.

These observations allow us to say that the alkaline treatment increased the availability of functional groups on the biosorbent surface to interact with heavy-metal ions from aqueous media, increasing its efficiency. Unfortunately, the predominantly electrostatic nature of the interactions that take place in the biosorption processes makes the geometric dimension of the metal ions play an important role, which significantly reduces the selectivity of this biosorption process.

However, the biosorption capacity of Na-SW biosorbent for Pb(II), Cd(II), and Zn(II) is comparable with the values reported in the literature for other biosorbents (Table 3), highlighting the potential of this material to be used in the removal processes of metal ions from aqueous effluents.

### 3.3. Effect of Contact Time on Removal Efficiency and Kinetic Modeling

**Figure 5:**
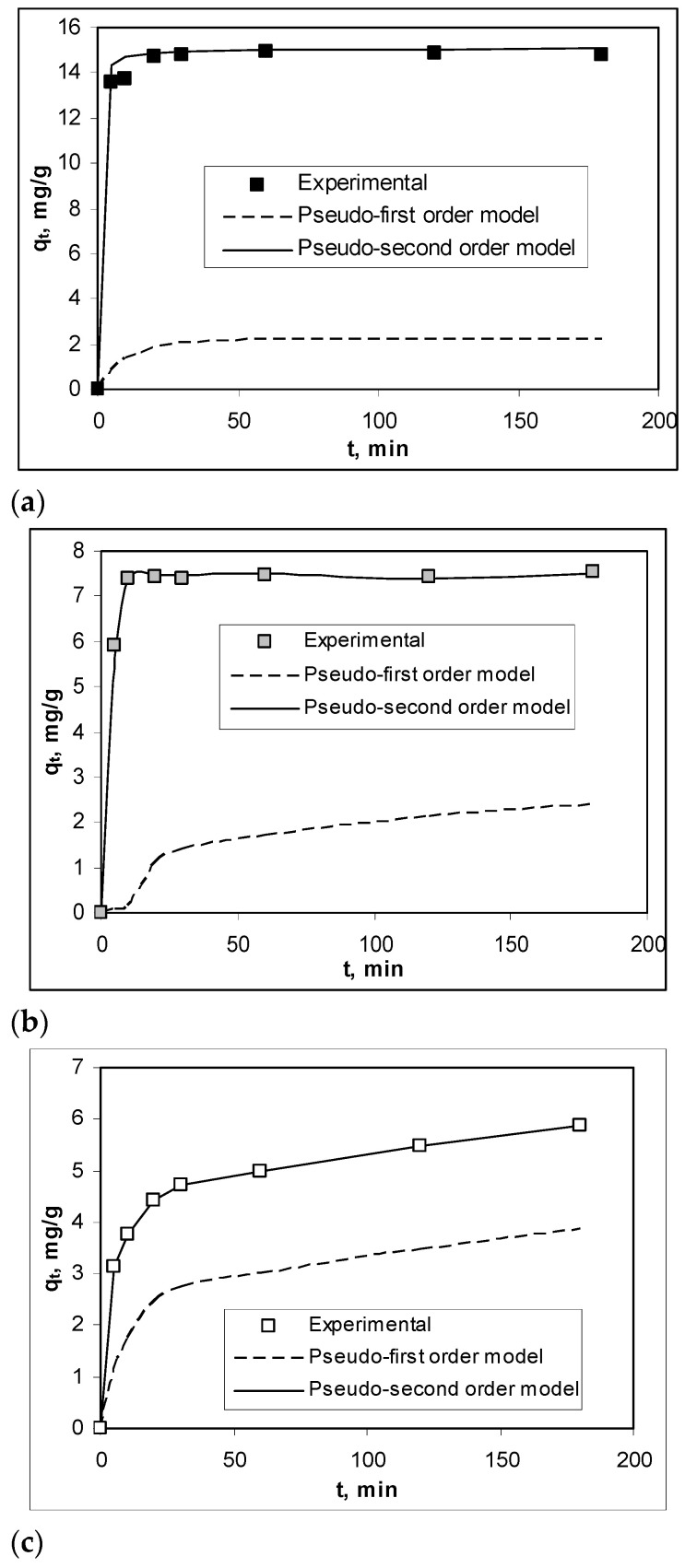
Experimental and modeled kinetic curves obtained for the biosorption of Pb(II) (**a**), Cd(II) (**b**), and Zn(II) (**c**) on Na-SW biosorbent.

In order to highlight the practical applicability of the Na-SW biosorbent in the removal of Pb(II), Cd(II), and Zn(II) ions, it is necessary to examine the influence of contact time on the efficiency of the biosorption processes. The experimental results obtained in these experiments, illustrated in Figure 5, show that the contact time required to reach the equilibrium state was very short (maximum 10 min), and this value did not depend on the nature of the heavy-metal ions from aqueous solution. In this time interval (10 min), the retention of all metal ions was quantitative (over 93% in the case of Pb(II), 81% in the case of Cd(II), and 76% in the case of Zn(II), respectively), which is a real advantage from the perspective of using this biosorbent on a large scale. The quantitative description of the kinetics of the studied biosorption processes was determined by modeling the experimental data using pseudo-first-order and pseudo-second-order kinetic models. The kinetic curves obtained from the modeling are shown in Figure 5, while the calculated kinetic parameters are summarized in Table 3. As can be seen from Figure 5 and Table 4, the pseudo-second-order kinetic model described very well the biosorption of Pb(II), Cd(II), and Zn(II) on Na-SW biosorbent, because the regression coefficients (*R^2^*) were almost equal to 1, and the values of biosorption capacities calculated from this model (*q_e_*^calc^, mg/g) and those obtained experimentally (*q_e_*^exp^, mg/g) were very close.

Therefore, the retention of Pb(II), Cd(II), and Zn(II) ions from aqueous solution on Na-SW biosorbent takes place through physicochemical interactions, most likely electrostatic type, in which superficial functional groups of the biosorbent are involved. This possible interaction mechanism of the heavy-metal ions with the functional groups of Na-SW is also supported by the FTIR spectra recorded for the biosorbent before and after the metal ion biosorption.

**Figure 6:**
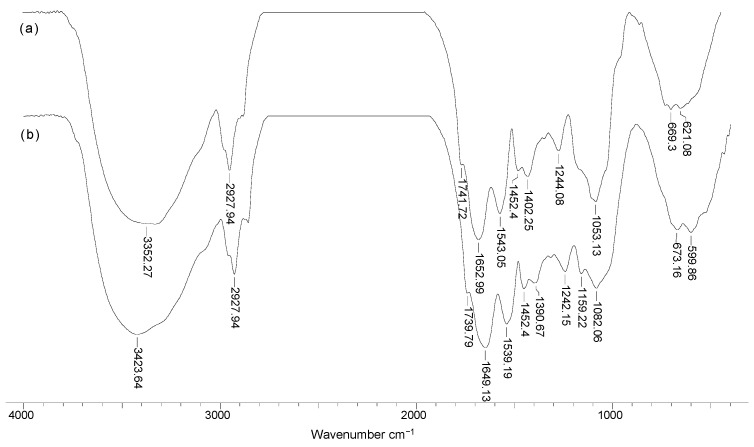
FTIR spectra of Na-SW biosorbent before (**a**) and after (**b**) Pb(II) ion biosorption from aqueous solution.

Figure 6 illustrates the FTIR spectra obtained in the case of Pb(II) ion biosorption on Na-SW biosorbent. A careful analysis of these spectra (Figure 6) reveals that, after the retention of Pb(II) ions (spectrum b), the spectral shape did not change significantly (no new absorption bands appeared), with only small displacements of the maximum wave numbers (compared to spectrum a). Therefore, the biosorption of heavy-metal ions did not change the structure of the superficial functional groups of Na-SW biosorbent, but only changed their chemical vicinity, mostly by breaking some physical bonds.

Breaking the physical bonds in the superficial structure of the biosorbent during the biosorption process is most likely a reversible process, whereby, once the heavy metal ions are removed by desorption, they are restored again and the biosorbent can be reused in another biosorption cycle.

### 3.4. Desorption of Heavy-Metal Ions and Biosorbent Regeneration

Desorption of heavy-metal ions (Pb(II), Cd(II), and Zn(II)) from Na-SW was tested in three biosorption/desorption cycles, and the experiments were performed for each metal ion, using the same sample of biosorbent. Considering the nature of the superficial functional groups of Na-SW involved in the biosorption process and the strong acidic characteristic of HNO_3_, a 10^−2^ N HNO_3_ solution was used as the desorption agent. Thus, each sample of Na-SW loaded with metal ions (0.1 g) was treated with 10 mL of a 10^−2^ N HNO_3_ solution and left for 3 h on each occasion.

**Figure 7:**
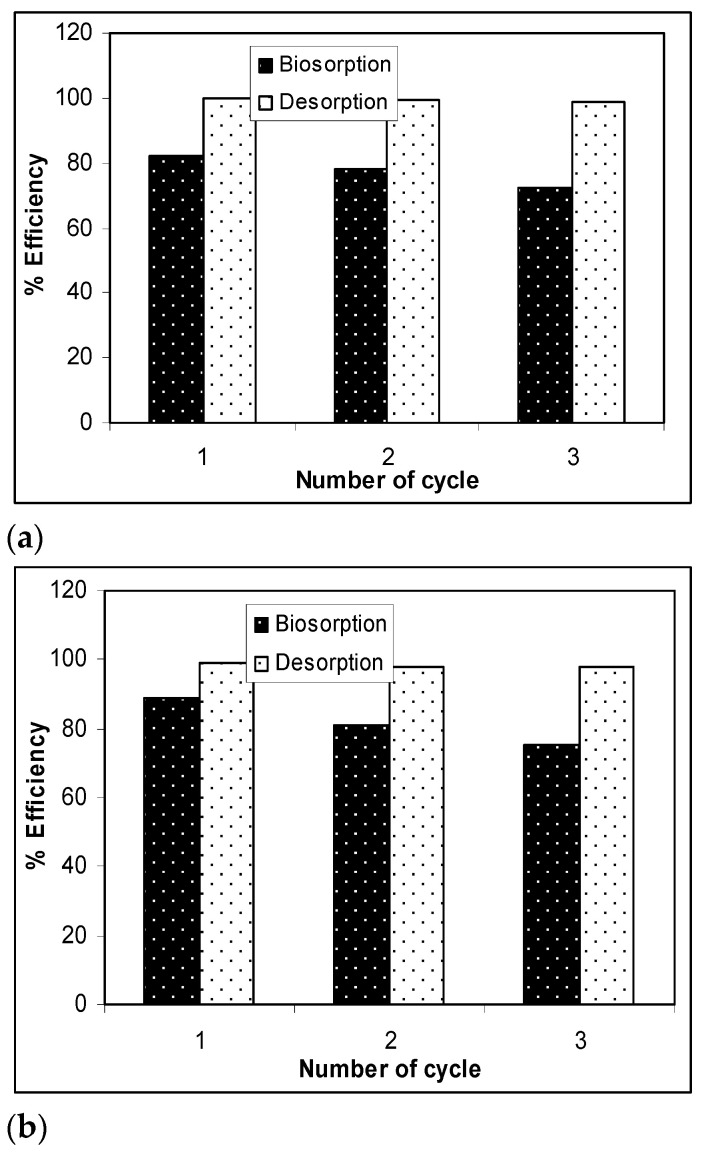

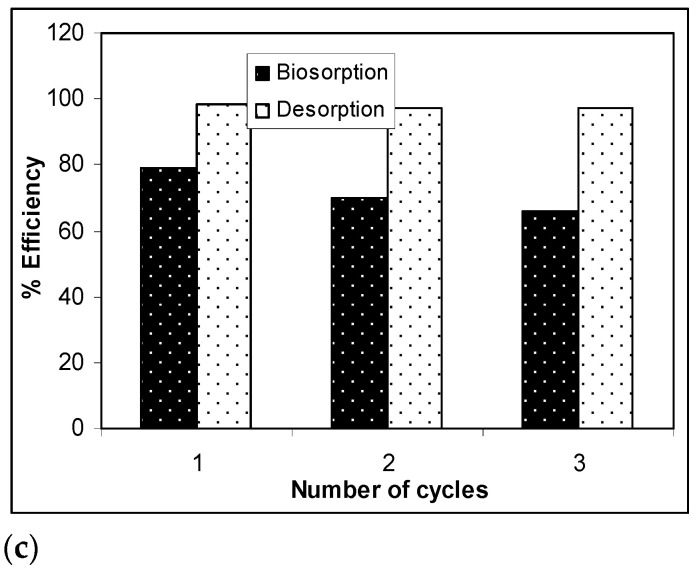
Biosorption/desorption efficiency of heavy metal ions on Na-SW biomass during the three cycles: (**a**) Pb(II); (**b**) Cd(II); (**c**) Zn(II).

The results presented in Figure 7 show that, after three desorption/biosorption cycles, the biosorption capacity of Na-SW slightly decreased for each metal ion, while the metal ion desorption was quantitative and did not seem to be influenced by the number of cycles of biosorbent use. Specifically, the biosorption efficiency decreased by 10% in the case of Pb(II), 12% in the case of Cd(II), and 14% in the case of Zn(II) over the three cycles, whereas the decrease in desorption efficiency was much lower (below 2%) for all studied heavy-metal ions. These experimental results demonstrate that a 10^−2^ N HNO_3_ solution allowed the quantitative recovery of retained metal ions (>97%) and ensured the efficient regeneration of the Na-SW biosorbent, which could be used again after a simple washing and drying step.

### 3.5. Practical Applicability of Na-SW Biosorbent in the Treatment of Wastewater

The practical applicability of the Na-SW biosorbent in the removal of Pb(II), Cd(II), and Zn(II) ions from aqueous effluents was tested using simulated laboratory wastewater samples obtained from tap water, adjusting the initial concentration of metal ions to a given value, while the initial pH and biosorbent dosage were maintained constant at optimal values (pH = 3.4; 5 g of biosorbent/L). Some important parameters of the artificial wastewater before and after biosorption of each studied heavy-metal ion on Na-SW, determined according to standard procedures [31], are summarized in Table 5.

It can be observed from Table 4 that, after the treatment of simulated wastewater with Na-SW biosorbent, the concentration of all heavy-metal ions decreased significantly (over 70%), while the values of other parameters remained practically unchanged. Two aspects should be highlighted according to the data presented in Table 4. The first is that, after biosorption, the pH of treated wastewater increased to 5.5, but this value remained below the maximum permissible limit (6.5–8.5) [42].

This means that, after biosorption, the treated wastewater must still be neutralized before it is discharged into the environment. The second aspect is related to the oxidability index (CCO, mg O_2_/L) which remained almost constant after biosorption processes. The constant value of this parameter, before and after biosorption, indicates that the Na-SW biosorbent is stable in aqueous solution and does not release organic compounds from its composition that could contaminate the treated effluents.

### 3.6. Future Research Plan for the Recovery of Metal Ions from Industrial Effluents

Metal ions are important raw materials for the economy of any country and, for this reason, the need for such materials must be ensured for the production of key components of different products [43,44]. Therefore, the recovery of metal ions from various wastes, considered as secondary resources, is becoming increasingly important in today’s society [45,46].

**Figure 8:**
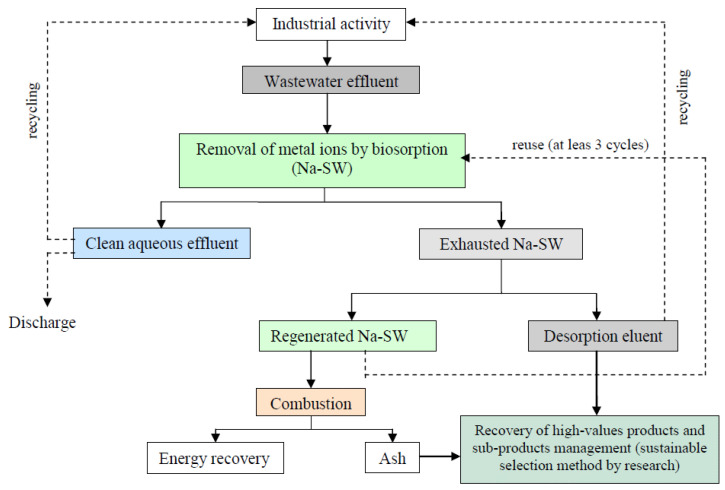
Pathways for the recovery of metals from exhausted biomass and effluent applied for the removal of heavy metals from wastewater using Na-SW biosorbent.

In this context, the exhausted biomass and the effluent resulting from the desorption stage can be considered as secondary sources of metal ions. Thus, wastewater treatment by biosorption of metal ions can be integrated with the valorization of the exhausted biomass as a subeconomic source for metal recovery, using them as secondary raw materials. Figure 8 illustrates the pathways for the recovery of metal ions from industrial effluents, taking into account the experimental results presented in previous sections. This solution is linked to the concept of the circular economy and has the potential to generate new business opportunities aimed at recovering high-value products, ensuring their use as raw materials. The metal ions contained in exhausted Na-SW biosorbent or in desorption eluent can be recovered via two specific procedures (Figure 8):

(i). in the case of exhausted Na-SW—thermal combustion of biomass and separation of metals from the resulting ash by sustainable procedures (such as (bio)leaching or (bio)extraction), together with the evaluation of the energy that can be recovered from the combustion process;
(ii). in the case of desorption eluent—two possibilities can be identified, either the recovery of metal ions from this eluent via well-known processes (electrochemical or electro-driven processes or microbial electro-metallurgy [47,48]) or the reuse of the desorption eluent as it is in industrial activities as a source of metal ions.

The applicability of either of these procedures will depend on economic and ecological feasibility results, which will be presented in future studies.

## 4. Conclusions

In this study, the soy waste biomass resulting from oil extraction was treated with alkaline solution, and the obtained material (Na-SW) was used as biosorbent for the removal of Pb(II), Cd(II), and Zn(II) ions from aqueous media. In this way, we wanted to determine the potential of exploiting these biomass wastes in the environmental cleaning processes, in accordance with the principles of the circular economy. The biosorptive performance of Na-SW biosorbent was examined in batch experiments as a function of initial metal ion concentration and contact time, in optimal experimental conditions (pH of 3.4; 5 g of biosorbent/L). The experimental isotherms were well described by the Langmuir model, indicating that the retention of Pb(II), Cd(II), and Zn(II) was through monolayer biosorption. The maximum biosorption capacities in monocomponent systems followed the order Zn(II) (0.49 mmol/g) > Cd(II) (0.41 mmol/g) ≈ Pb(II) (0.40 mmol/g), which is similar to the variation in ionic radius of these ions. The kinetic modeling of experimental data indicated that the pseudo-second-order model was the most appropriate to describe the biosorption processes. The regeneration of the biosorbent and quantitative recovery of retained metal ions could be done with a 10^−2^ N HNO_3_ solution, and the biosorptive performance of Na-SW did not change significantly after three biosorption/desorption cycles. The practical applicability of the Na-SW biosorbent for the removal of Pb(II), Cd(II), and Zn(II) ions from aqueous effluents was highlighted using simulated wastewater samples. Over 70% of the initial heavy-metal ions were removed by biosorption, while the values of the other quality parameters remained almost unchanged. Therefore, Na-SW can be considered an efficient biosorbent that can be used for the removal of heavy-metal ions for industrial applications and as a subeconomic source of heavy metals as secondary raw materials. Lastly, a future research plan for the recovery of metal ions from industrial effluents, in accordance with the principles of the circular economy, was designed to highlight the practical applicability of this study on a large scale.

## Figures and Tables

**Table 1:** Spectrophotometric methods used for the analysis of heavy-metal ions [30].

Metal Ion	Color Reagent	λ (nm)	Experimental Conditions
Pb(II)	4-(2-pyridylazo)-resorcinol	530	pH = 10; blank solution
Cd(II)	Xylenol Orange	575	pH = 6.0; blank solution
Zn(II)	Xylenol Orange	570	pH = 6.0; distilled water

**Table 2:** Isotherm parameters obtained for the biosorption of studied metal ions on Na-SW biosorbent.

Isotherm Model	Parameter	Pb(II)	Cd(II)	Zn(II)
Langmuir model $\frac{1}{q}=\frac{1}{q_{\max}}+\frac{1}{q_{\max}\cdot K_{L}}\cdot \frac{1}{c}$	*R^2^*	0.9981	0.9913	0.9928
	*q*_max_, mg/g	84.03	45.97	32.39
	*K_L_*, L/g	0.0047	0.0047	0.0041
Freundlich model $\ln q=\ln K_{F}+\frac{1}{n}\ln c$	*R^2^*	0.8492	0.6966	0.9136
	1/*n*	0.7823	0.7420	0.7188
	*K_F_*, L/g	0.6761	0.9612	0.1209

Notations: *q*—biosorption capacity at equilibrium, *q*_max_—maximum biosorption capacity, *K_L_*—Langmuir constant; *K_F_*—Freundlich constant, *n*—heterogeneity factor [33].

**Table 3:** Comparison of the maximum biosorption capacity of Na-SW biosorbent for studied metal ions with different biomass-based biosorbents.

Biosorbent	Pb(II)	Cd(II)	Zn(II)	References
*Laminaria hyperborea* (brown algae)	30.49	52.39	19.26	[34]
*Sargassum* sp. (seaweed)	139.11	61.19	28.89	[35]
non-living *Pseudomonas* strains	43.57	-	18.49	[36]
*Geobacillus thermodenitrificans*	36.28	35.57	24.03	[37]
Lignin	89.45	19.34	14.82	[38]
Coconut shell	54.61	11.92	17.14	[39]
Mustard waste biomass	73.78	26.94	18.59	[40]
Na-SW biosorbent	84.03	45.97	32.39	This study

**Table 4:** Kinetic parameters obtained for the biosorption of Pb(II), Cd(II), and Zn(II) on Na-SW biosorbent.

Kinetic Model [41]	Parameter	Pb(II)	Cd(II)	Zn(II)
	*q_e_*^exp^, mg/g	14.84	7.56	5.07
Pseudo-first-order model $\ln(q_{e}-q_{t})=\ln q_{e}-k_{1}\cdot t$	*R^2^*	0.8701	0.8612	0.8819
	*q_e_*^calc^, mg/g	4.19	3.34	2.97
	*k*_1_, 1/min	0.0406	0.0540	0.0572
Pseudo-second-order model $\frac{t}{q_{t}}=\frac{1}{k_{2}\cdot q_{e}^{2}}+\frac{t}{q_{e}}$	*R^2^*	0.9999	0.9999	0.9999
	*q_e_*^calc^, mg/g	15.08	7.56	5.15
	*k*_2_, g/mg min	0.4456	0.5275	0.5881

Notations: *q_e_, q_t_*—biosorption capacity at equilibrium and at time t; *k*_1_—rate constant of pseudo-first-order kinetic equation; *k*_2_—pseudo-second-order rate constant.

**Table 5:** Parameters of the artificial wastewater before and after the treatment with Na-SW biosorbent.

Parameter	Initial	After Biosorption		
		Pb(II)	Cd(II)	Zn(II)
Pb(II), mg/L	41.64	0.56	-	-
Cd(II), mg/L	23.05	-	0.16	-
Zn(II), mg/L	26.37	-	-	0.27
pH	3.40	5.48	5.56	5.52
CCO, mg O_2_/L	112.03	118.16	120.23	118.65
Cl^−^, mg/L	72.16	73.02	72.87	72.61
NO_3_^−^, mg/L	16.04	15.89	16.23	15.86
Ca(II), mg/L	89.14	94.01	93.68	94.54
Mg(II), mg/L	65.12	66.02	66.32	66.14

## Data Availability

Not applicable.
